# Janus Kinase and Interleukin-6 Inhibitor-Induced Lipid Modulation and Its Association With Major Adverse Cardiovascular Events in Rheumatoid Arthritis: A Systematic Review of Clinical and Biomarker Evidence

**DOI:** 10.7759/cureus.106584

**Published:** 2026-04-07

**Authors:** Rohan M Patel, Lila Dudley, Mackenzie Morse, Christos G Mihos, Marc M Kesselman

**Affiliations:** 1 Osteopathic Medicine, Nova Southeastern University Dr. Kiran C. Patel College of Osteopathic Medicine, Fort Lauderdale, USA; 2 Cardiology, Mount Sinai Heart Institute, Columbia University College of Physicians and Surgeons, Miami Beach, USA; 3 Rheumatology, Nova Southeastern University Dr. Kiran C. Patel College of Osteopathic Medicine, Fort Lauderdale, USA

**Keywords:** disease-modifying anti-rheumatic drugs, il6 inhibition, inflammatory markers, jak inhibitor, ldl cholesterol, lipid metabolism, lipid paradox, major adverse cardiovascular events, rheumatoid arthritis, total cholesterol

## Abstract

Rheumatoid arthritis (RA) is a chronic systemic inflammatory disease associated with increased cardiovascular (CV) risk and alterations in lipid metabolism. Janus kinase inhibitors (JAKi) and interleukin-6 inhibitors (IL-6i) have offered effective control of disease activity; however, their impact on lipid profiles and subsequent CV outcomes has yet to be fully elucidated. This systematic review aimed to evaluate the effects of JAKi and IL-6i on lipid profiles, inflammatory biomarkers, and major adverse cardiovascular events (MACE) in patients with RA. Nineteen peer-reviewed studies published after 2015 were identified that examined the influence of these targeted therapies on laboratory parameters and/or MACE. The findings revealed that both approaches were associated with reductions in erythrocyte sedimentation rate (ESR), C-reactive protein (CRP), and high-sensitivity CRP (hsCRP), although the magnitude of change varied substantially. Most studies reported an increase in total cholesterol and low-density lipoprotein cholesterol (LDL-C), often accompanied by modest increases in high-density lipoprotein cholesterol (HDL-C) following treatment. Despite these lipid changes, the incidence of MACE generally remained low. IL-6i therapy showed no significant increase in absolute CV events, whereas some JAKi studies reported numerically higher rates of MACE. These findings suggest that lipid alterations observed with targeted RA therapies may reflect the complex relationship between inflammation and lipid metabolism, often referred to as the “lipid paradox,” where systemic inflammation in RA is associated with lower circulating cholesterol levels despite increased CV risk. In this context, the increases in lipid levels observed after effective RA therapy may actually reflect normalization of lipid metabolism rather than necessarily reflecting an increase in CV risk. However, evidence from randomized trials such as the ORAL Surveillance Trial has demonstrated increased MACE risk with tofacitinib in higher-risk populations, and heterogeneity across observational studies limits causal interpretation. Further prospective studies are required to assess whether JAKi and IL-6i-induced lipid modulation actually translates into meaningful differences in CV outcomes. Until additional evidence is available, optimal inflammatory control and guideline-directed lipid management remain essential components of CV risk reduction in RA.

## Introduction and background

Rheumatoid arthritis (RA) is a chronic autoimmune condition characterized by systemic inflammation, with a predilection for synovial joints [1]. Early in the disease course, RA usually negatively impacts the metacarpophalangeal, interphalangeal, wrist, and knee joints [2]. Patients typically present with progressive manifestations, including bilateral joint stiffness in their extremities and range of motion deficits after long periods of rest, characteristically in the morning. Over time, patients often report systemic symptoms, including fever, fatigue, malaise, and weight loss. Methotrexate, a conventional synthetic disease-modifying anti-rheumatic drug (csDMARD), is the standard therapeutic agent used as the initial monotherapy for RA [3]. If csDMARD monotherapy is inadequate, patients should be escalated to biologic DMARDs (bDMARDs) or targeted synthetic DMARDs (tsDMARDs), including agents, such as tumor necrosis factor (TNF)-α inhibitors, interleukin inhibitors (IL-6i), rituximab, or Janus kinase inhibitors (JAKi) [4].

The pathophysiology of RA is commonly tied to increased levels of pro-inflammatory cytokines, including TNF-α, IL-6, and IL-1, which play a pivotal role in accelerating atherosclerosis and subsequent vascular dysfunction. This chronic inflammatory state promotes endothelial injury, oxidative stress, and plaque instability, all of which contribute to a greater risk of cardiovascular (CV) disease development [5]. Patients with RA frequently exhibit both traditional CV risk factors, including hypertension, diabetes, and dyslipidemia, as well as disease-specific inflammatory mechanisms that accelerate atherosclerosis. This chronic systemic inflammation and cytokine-driven endothelial dysfunction further contribute to CV disease in this population, making the CV effects of targeted biologic therapies an area of growing clinical interest [6]. A significant cause of morbidity and mortality in patients with RA is major adverse cardiovascular events (MACE), which may include cerebrovascular accident (CVA), myocardial infarction (MI), and CV death [7]. The prevalence of CV events in patients with RA is nearly 1.5 times higher than in the overall population; this is attributed to an accelerated progression of atherosclerosis secondary to chronic inflammation, adverse effects of RA therapy, and inability to adequately prevent atherosclerotic plaque progression in patients with RA [8]. Despite the augmented CV risk in RA, traditional lipid laboratory findings are often deceptively low or normal, likely due to inflammation-driven cytokine effects on hepatic lipid metabolism, resulting in reduced circulating lipid concentrations. This counterintuitive observation has given rise to the "lipid paradox," wherein patients with clinically active RA may exhibit lower low-density lipoprotein cholesterol (LDL-C) and total cholesterol levels, while still facing an increased risk of adverse CV events [9]. 

JAKs are cytoplasmic non-receptor tyrosine kinases that regulate gene transcription of numerous inflammatory molecules, thereby furthering the clinical severity of conditions such as RA, hyperlipidemia, and cancer [10]. JAKi, including tofacitinib, have been approved as an adjunct therapy to prevent inflammatory disease progression. Although JAKi have been effective in reducing inflammation tied to RA, these agents have also been linked to changes in lipid function and raised the question of elevated CV disease risk. Recent comparative safety studies have examined CV outcomes among different classes of targeted therapies for RA. Large observational registry analyses have reported differences in CV risk between JAKi, TNF-α inhibitors, and IL-6i. The ORAL Surveillance trial in patients with RA aged ≥ 50 years with at least one CV risk factor demonstrated higher rates of MACE with tofacitinib compared with TNF-α inhibitors, suggesting that reductions in systemic inflammation may be counterbalanced by adverse effects on lipid metabolism, endothelial function, and prothrombotic pathways [11]. Of note, however, participants were aged ≥ 50 years and had at least one cardiometabolic risk factor, and therefore the observed risk may not be fully generalizable to lower-risk populations [12]. For this reason, regulatory agencies have issued warnings for certain JAKi, advising caution in patients with a history of CV disease or risk factors [13]. 

IL-6 is a cytokine present in synovial fibroblasts, activated macrophages, and lymphocytes. By initiating the production of acute-phase reactants, IL-6 plays a pivotal role in the initiation and advancement of the inflammatory cascade. Concurrently, IL-6 activates endothelial cells to promote the expression of adhesion molecules, encouraging potential vascular insufficiency [14]. Given these pathophysiological effects, IL-6i, including tocilizumab, have been approved as an adjunct treatment for patients with chronic inflammatory diseases. Although these agents have proven to be effective in reducing inflammation, there is some concern surrounding CV risk. IL-6 has been shown to be involved in adipose metabolism, in which elevated levels of IL-6 have been associated with increased coronary artery calcium scores [15]. However, a consequence of IL-6i therapy is often an increase in total cholesterol, which has been associated with a decrease or no significant change in CV risk in patients with RA, validating the aforementioned "lipid paradox” [16-17]. 

In patients with RA with an unsuccessful therapeutic response to methotrexate monotherapy, adding a JAKi or IL-6i may be beneficial. Although these targeted agents can significantly reduce inflammation and improve outcomes, their respective impacts on lipid profiles and subsequent CV risk have raised questions regarding their safety. This review aimed to examine adult patients with clinically diagnosed RA who were treated with an IL-6i or JAKi, with or without concurrent methotrexate, corticosteroid, or DMARDs. The goal was to further our understanding of the cardiometabolic and lipid-related consequences of IL-6i and JAKi therapy in this population. Ultimately, these findings could guide future prospective studies that help establish guidelines for clinicians, who must balance nuanced inflammatory control with CV safety when selecting targeted therapies for RA based on complex patient profiles with accompanying comorbidities and risk factors.

## Review

Materials and methods

Eligibility Criteria

This review focused on adult patients (≥18 years) diagnosed with RA, as defined by the original studies. No restrictions were placed on disease duration, baseline disease activity, or CV risk status. Interventions of interest included treatment with JAKi or IL-6i, including but not limited to tofacitinib, baricitinib, upadacitinib, filgotinib, tocilizumab, and sarilumab. Comparator groups included baseline lipid and/or inflammatory biomarker measurements, placebo groups, or untreated controls, as reported in individual studies. Outcomes of interest included lipid parameters (LDL-C, HDL-C, total cholesterol, lipoprotein(a) (Lp(a))), inflammatory biomarkers (C-reactive protein (CRP), high-sensitivity CRP (hsCRP), erythrocyte sedimentation rate (ESR)), and CV outcomes, including MACE (Table 1). Studies were eligible if they evaluated adult patients with diagnosed RA; assessed exposure to a JAKi or IL-6i with or without concurrent baseline methotrexate, corticosteroid, or DMARD therapy; reported lipid parameters, inflammatory biomarkers, or CV outcomes; and were randomized controlled trials, cohort studies, or observational studies. Exclusion criteria included case reports, reviews, editorials, non-English publications, and studies without original data.

**Table 1:** PICO framework IL-6: interleukin-6; JAK: Janus kinase; MACE: major adverse cardiovascular events; LDL-C: low-density lipoprotein cholesterol; HDL-C: high-density lipoprotein cholesterol; Lp(a): lipoprotein a; hsCRP: high-sensitivity C-reactive protein; CRP: C-reactive protein; ESR: erythrocyte sedimentation rate; PICO: population/patient, intervention, comparison, and outcome

PICO framework for systematic reviews	
Population	Patients ≥ 18 years with clinically diagnosed rheumatoid arthritis
Intervention	Treatment with IL-6 inhibitors or JAK inhibitors with or without baseline methotrexate, corticosteroids, or disease-modifying anti-rheumatic drugs
Comparison	Baseline lipid and inflammatory biomarker levels, or placebo/untreated control groups (when available)
Outcome	Incidence of MACE, laboratory fluctuations in lipid parameters (LDL-C, HDL-C, total cholesterol, Lp(a)), or fluctuations in inflammatory biomarkers (hsCRP/CRP, ESR)

Search Strategy

A comprehensive literature search was conducted in EMBASE, Web of Science, and OVID Medline to identify peer-reviewed studies. These databases were accessed through Nova Southeastern University’s library subscription. Search terms included MeSH and free-text keywords related to RA, JAKi, IL-6i, lipid parameters, and CV outcomes (Appendices A-C).

Selection Process

**Figure 1:**
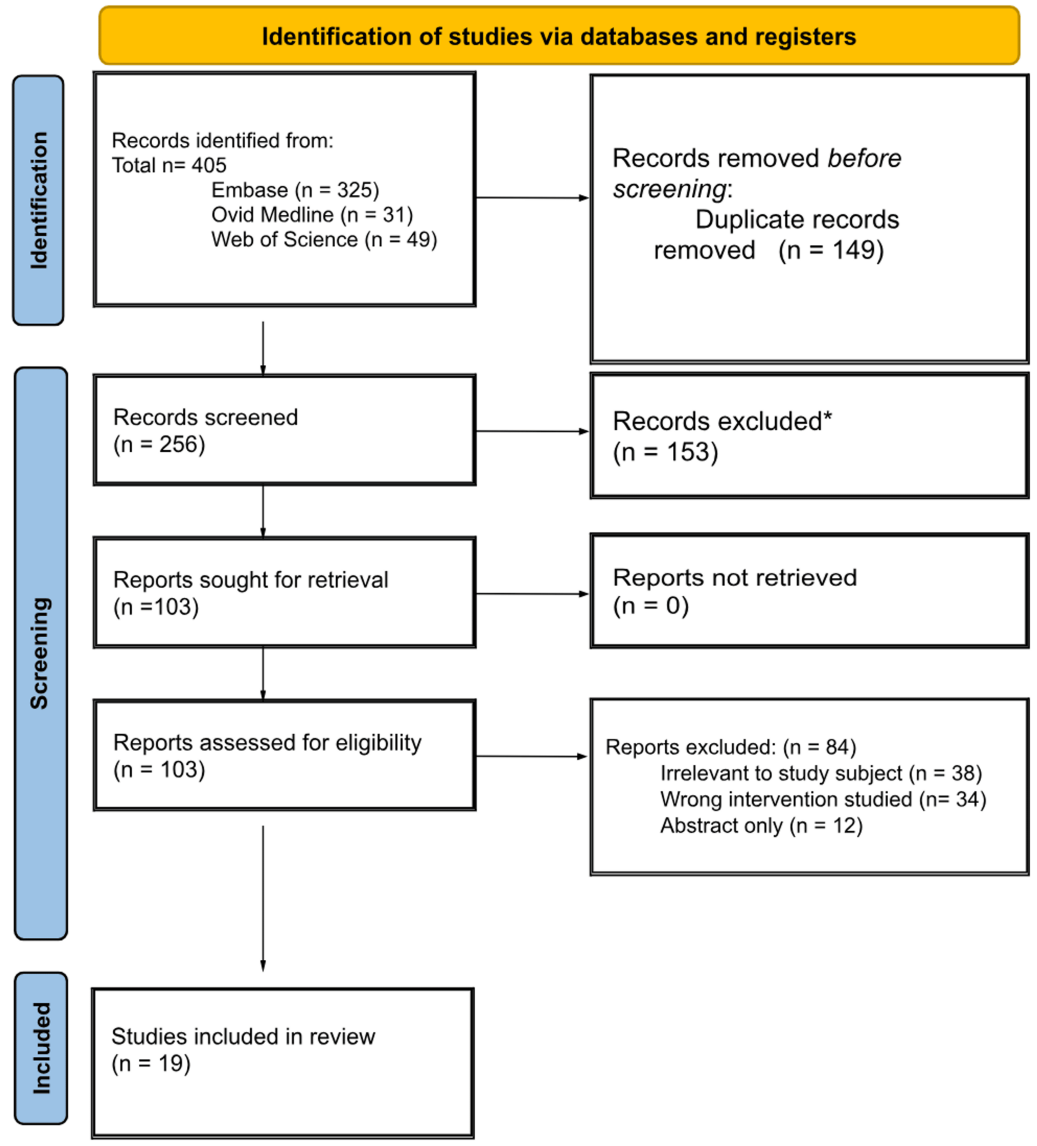
Preferred Reporting Items for Systematic Reviews and Meta-Analyses (PRISMA) flow diagram *Ineligible by date of publication, publication type, and study design

All retrieved articles (n=405) were uploaded into the Rayyan systematic review collaboration software (Rayyan Systems; Cambridge, Massachusetts, USA). After removing 149 duplicates, two reviewers (RP and LD) independently screened titles and abstracts against the inclusion criteria, excluding 153 studies. One-hundred three full-text articles were assessed, and 84 were subsequently excluded for the following reasons: irrelevant to the study subject (n=38), wrong intervention (n=34), or abstract only (n=12). Nineteen studies met the eligibility criteria and were included in the review. The study selection process followed PRISMA guidelines, and the flow diagram is presented in Figure 1 [18]. Any discrepancies were solved through discussion with a third reviewer (MM).

Data Collection Process

Data were extracted using a standardized data chart in Google Sheets (Google Corporation; Mountain View, CA, USA). Variables collected included study design, publication year, sample size, interventions, comparators, and outcomes (lipid and inflammatory biomarkers, MACE). MACE was defined according to the endpoints reported in the individual studies. In CV research, MACE most commonly refers to a three-point composite of nonfatal MI, CVA, and CV death. Some studies even use expanded definitions (four-point MACE) that include additional events such as unstable angina or coronary revascularization [19]. When provided, study-specific MACE definitions were recorded and summarized (Table 2). For studies that reported MACE without specifying component events, the outcome was reported as described by the authors without further reclassification. Three reviewers (RP, LD, and MM) initially divided the studies and independently performed data extraction for their assigned studies. Subsequently, each reviewer cross-checked the data extracted by the others to ensure accuracy, and any discrepancies were resolved through discussion. Where data were missing or unclear, attempts were not made to contact the study authors.

**Table 2:** Study-specific MACE definitions MACE: major adverse cardiovascular events; MI: myocardial infarction; CVA: cerebrovascular accident; CV: cardiovascular; N/A: the study did not provide a definition of MACE with composite endpoints

Author, year	Study-specific MACE definition (if provided)
Aletaha et al., 2021 [20]	MI and CVA
Alsulaim et al., 2021 [21]	Stable angina, unstable angina, nonfatal MI, or CVA
Anyfanti et al., 2024 [22]	N/A
Benucci et al., 2023 [23]	N/A
Charles-Schoeman et al., 2023 [24]	N/A
Charles-Schoeman et al., 2019 [25]	MI, CVA, or CV death
Charles-Schoeman et al., 2016 [26]	CV non-fatal (MI and CVA) and CV fatal (coronary, cerebrovascular, cardiac, non-cardiac vascular)
Cohen et al., 2021 [27]	Non-fatal MI, non-fatal CVA, or CV death
Czókolyová et al., 2022 [28]	N/A
Dougados et al., 2017 [29]	MI, CVA, or CV death
Gentileschi et al., 2024 [30]	N/A
Gerasimova et al., 2024 [31]	N/A
Molander et al., 2023 [32]	N/A
Novikova et al., 2019 [33]	N/A
Pierini et al., 2021 [34]	N/A
Rao et al., 2015 [35]	Definite or probable nonfatal MI, nonfatal CVA, or CV death
Smolen et al., 2019 [36]	Non-fatal MI, non-fatal CVA, and CV death
Taylor et al., 2019 [37]	MI, CVA, or CV death
Yoshida et al., 2023 [38]	Non-fatal MI, non-fatal CVA, or CV death

Synthesis Methods

Due to heterogeneity in study designs, populations, interventions, and specific outcome definitions, a quantitative meta-analysis was not performed. Results were synthesized using a narrative approach, within a systematic review framework, summarizing outcomes qualitatively in the text. Lipid values reported in mmol/L were converted to mg/dL for consistency using standard conversion factors, whereas CRP and hsCRP values were standardized to mg/L to allow for descriptive comparison across studies. MACE outcomes were reported according to the definitions used in the original studies without further standardization. Summary statistics varied across studies as well, with some reporting means with standard deviations and others reporting medians with IQRs. Because of these incompatible dispersion metrics, quantitative pooling of lipid and inflammatory marker values was not performed. 

Risk of Bias Assessment 

Risk of bias was independently assessed by two reviewers using the Cochrane Risk-of-Bias Tool for RCTs Version 2 (RoB 2) and the Newcastle-Ottawa Scale (NOS) for observational studies [39,40]. Each study received an overall judgment of low risk, some concerns (RoB 2)/moderate risk (NOS), or high risk of bias. Domain-level findings were combined, according to their respective frameworks, to enhance interpretability and organization (Table 3).

**Table 3:** Risk of bias assessment using the Cochrane risk-of-bias tool for randomized control trials (RCTs) version 2 (RoB 2) or the Newcastle-Ottawa Scale (NOS) based on study design RoB 2 was used for RCTs. NOS was used for observational studies. N/A: bias assessment does not apply to the selected study

Risk of bias assessment									
Author, year	Scale used	Comparability	Outcome	Randomization process	Deviations from intended interventions	Measurement of outcome	Missing outcome data	Selection of the reported result	Overall risk of bias
Aletaha et al., 2021 [20]	Newcastle-Ottawa	1	2	3	N/A	N/A	N/A	N/A	Moderate risk
Alsulaim et al., 2021 [21]	Newcastle-Ottawa	2	3	3	N/A	N/A	N/A	N/A	Low risk
Anyfanti et al., 2024 [22]	Newcastle-Ottawa	2	3	2	N/A	N/A	N/A	N/A	Low risk
Benucci et al., 2023 [23]	Newcastle-Ottawa	2	3	3	N/A	N/A	N/A	N/A	Low risk
Charles-Schoeman et al., 2023 [24]	Newcastle-Ottawa	2	3	4	N/A	N/A	N/A	N/A	Low risk
Charles-Schoeman et al., 2019 [25]	Cochrane RoB	N/A	N/A	Low risk	Low risk	Low risk	Some concerns	Low risk	Some concerns
Charles-Schoeman et al., 2016 [26]	Cochrane RoB	N/A	N/A	Low risk	Low risk	Low risk	Some concerns	Low risk	Some concerns
Cohen et al., 2021 [27]	Cochrane RoB	N/A	N/A	Low risk	Low risk	Low risk	Low risk	Low risk	Low risk
Czokolyova et al., 2022 [28]	Newcastle-Ottawa	1	2	3	N/A	N/A	N/A	N/A	Moderate risk
Dougados et al., 2017 [29]	Cochrane RoB	N/A	N/A	Low risk	Low risk	Low risk	Low risk	Some concerns	Some concerns
Gentileschi et al., 2024 [30]	Newcastle-Ottawa	1	1	3	N/A	N/A	N/A	N/A	Moderate risk
Gerasimova et al., 2024 [31]	Cochrane RoB	N/A	N/A	Some concerns	Low risk	Low risk	Low risk	Low risk	Some concerns
Molander et al., 2023 [32]	Newcastle-Ottawa	1	1	4	N/A	N/A	N/A	N/A	Moderate risk
Novikova et al., 2019 [33]	Newcastle-Ottawa	1	2	2	N/A	N/A	N/A	N/A	Moderate risk
Pierini et al., 2021 [34]	Newcastle-Ottawa	2	2	3	N/A	N/A	N/A	N/A	Low risk
Rao et al., 2015 [35]	Newcastle-Ottawa	2	3	3	N/A	N/A	N/A	N/A	Low risk
Smolen et al., 2019 [36]	Cochrane RoB	N/A	N/A	Low risk	Low risk	Low risk	Low risk	Low risk	Low risk
Taylor et al., 2019 [37]	Newcastle-Ottawa	1	2	3	N/A	N/A	N/A	N/A	Moderate risk
Yoshida et al., 2023 [38]	Newcastle-Ottawa	1	3	3	N/A	N/A	N/A	N/A	Low risk

Protocol Registration

A protocol for this systematic review was not prospectively registered in PROSPERO, but all aspects of the review, including eligibility criteria, search strategy, selection, data extraction, and synthesis, were predefined according to PRISMA guidelines, ensuring methodological comprehension [18]. 

Results

A total of 19 studies met the inclusion criteria, comprising over 100,000 patients with a clinical diagnosis of RA. The included studies were classified as randomized controlled trials (n=6), prospective cohort studies (n=5), and retrospective cohort studies (n=8) published after 2015. For the studies that evaluated IL-6i, the primary agents included were tocilizumab, sirukumab, and sarilumab. For the studies that evaluated JAKi, the primary drugs included were tofacitinib, baricitinib, upadacitinib, and filgotinib. Each included study utilized varied interventions and dosing regimens (Table 4). Although methotrexate was the most common concomitant therapy, some patients continued using pre-evaluation corticosteroids or DMARDs. Across the included studies, outcomes were evaluated with respect to the incidence of MACE occurrences, lipid profile changes, and/or the response of inflammatory biomarkers. 

**Table 4:** Immunomodulatory interventions and dosages/routes (if available) of included studies Note: All routes were oral unless stated otherwise. IL-6i: interleukin-6 inhibitor; JAKi: Janus kinase inhibitor; SC: subcutaneously; IV: intravenously

Author, year	Intervention, dosages, and frequency (if available)
Aletaha et al., 2021 [20]	IL-6i: sirukumab 100 mg SC every two weeks or sirukumab 50 mg SC every four weeks
Alsulaim et al., 2021 [21]	IL-6i: tocilizumab SC once weekly or 4-8 mg/kg IV every four weeks
Anyfanti et al., 2024 [22]	JAKi: upadacitinib 15 mg daily, tofacitinib 5 mg twice daily, or baricitinib 5 mg daily
Benucci et al., 2023 [23]	JAKi: filgotinib 200 mg daily
Charles-Schoeman et al., 2023 [24]	JAKi: upadacitinib 15 mg daily or upadacitinib 30 mg daily
Charles-Schoeman et al., 2019 [25]	JAKi: tofacitinib 5 mg twice daily or tofacitinib 10 mg twice daily
Charles-Schoeman et al., 2016 [26]	JAKi: tofacitinib 5 mg twice daily or tofacitinib 10 mg twice daily
Cohen et al., 2021 [27]	JAKi: upadacitinib 15 mg daily or upadacitinib 30 mg daily
Czókolyová et al., 2022 [28]	JAKi: tofacitinib 5 mg twice daily or tofacitinib 10 mg twice daily
Dougados et al., 2017 [29]	JAKi: baricitinib 2 mg daily or baricitinib 4 mg daily
Gentileschi et al., 2024 [30]	JAKi: tofacitinib, baricitinib, upadacitinib, or filgotinib
Gerasimova et al., 2024 [31]	IL-6i: tocilizumab 8 mg/kg IV every four weeks (later changed to 162 mg SC once a week in some patients)
Molander et al., 2023 [32]	IL-6i: tocilizumab or sarilumab JAKi: tofacitinib, baricitinib, or upadacitinib
Novikova et al., 2019 [33]	JAKi: tofacitinib 5 mg twice daily (escalated to 10 mg twice daily in some)
Pierini et al., 2021 [34]	IL-6i: tocilizumab 4 mg/kg IV every 4 weeks or tocilizumab 162 mg SC every week
Rao et al., 2015 [35]	IL-6i: tocilizumab 4 mg/kg IV every four weeks or tocilizumab 8 mg/kg IV every four weeks
Smolen et al., 2019 [36]	JAKi: upadacitinib 15 mg daily or upadacitinib 30 mg daily
Taylor et al., 2019 [37]	JAKi: baricitinib 2 mg or baricitinib 4 mg
Yoshida et al., 2023 [38]	IL-6i: tocilizumab 8 mg/kg IV every four weeks, tocilizumab 162 mg SC every two weeks, or sarilumab 200 mg SC every two weeks JAKi: baricitinib 2 mg (if renally impaired) or 4 mg daily, tofacitinib 5 mg daily (if renally impaired) or twice daily, upadacitinib 15 mg daily, filgotinib 100 mg daily (if renally impaired) or 200 mg daily

*Erythrocyte Sedimentation Rate (ESR)*

Baseline ESR varied considerably across the included studies. Anyfanti et al. reported a decrease in mean ESR from 22.5 ± 8.4 mm/h at baseline to 18.0 ± 10.4 mm/h secondary to three months of JAKi therapy (either upadacitinib, tofacitinib, or baricitinib), corresponding to a 20% reduction [22]. This finding, however, was insignificant (p=0.316). Rao et al. evaluated baseline mean ESR levels among patients treated with tocilizumab and stratified patients based on those who went on to develop MACE with those who did not [35]. Baseline mean ESR was higher among patients who later developed MACE, being 52.0 ± 27.1 mm/h (n=50), compared with those who did not develop MACE, being 46.1 ± 26.8 mm/h (n=3,936), although the authors did not note a statistical comparison between groups. 

Some studies reported median ESR values, rather than mean values. Pierini et al. observed a reduction in median ESR from 33.0 mm/h (interquartile range (IQR) 23.0-60.0) at baseline to 6.0 mm/h (IQR 2.0-17.0) after three months of tocilizumab therapy, corresponding to an 82% reduction (p<0.0001) [34]. In the study by Benucci et al., three months of filgotinib therapy was associated with a reduction in median ESR from 35.0 mm/h (IQR 18.0-48.0) at baseline to 15.0 mm/h (IQR 10.0-29.75), demonstrating a 57% reduction (p=0.0001) [23]. In a longer-term follow-up of the same study, six months of filgotinib therapy was associated with a reduction in median ESR to 13.0 mm/h (IQR 10.0-28.0), representing a roughly 63% decrease from baseline (p=0.0001). Gerasimova et al. evaluated long-term outcomes following tocilizumab therapy and observed a reduction in median ESR from 48.0 mm/h (IQR 30.0-68.0) at baseline to 5.0 mm/h (IQR 3.0-10.0) after 60 months of treatment, which the authors reported was a significant finding (p<0.01) [31]. Similarly, Novikova et al. reported a decrease in median ESR from 5.1 mm/h (IQR 4.6-6.1) at baseline to 3.1 mm/h (IQR 2.40-3.98) after 12 months of tofacitinib therapy, corresponding to a 39% reduction (p<0.001) [33]. 

Together, these studies demonstrate consistent reductions in ESR following treatment with both JAKi and IL-6i, although the magnitude of reduction varied across populations and follow-up durations. 

C-reactive Protein (CRP)/High-Sensitivity C-reactive Protein (hsCRP)

Included studies reported either hsCRP or standard CRP. Although hsCRP provides greater sensitivity, both markers reflect the same inflammatory status, allowing for descriptive synthesis across the studies [41]. Studies reporting mean hsCRP values observed baseline levels ranging from 2.0 to 18.0 mg/L [22,29,36]. Following three months of treatment with JAKi therapy (either upadacitinib, tofacitinib, or baricitinib), Anyfanti et al. did not observe any significant change in mean CRP levels (p=1.000) [22]. Rao et al. assessed baseline mean CRP among patients receiving tocilizumab and stratified findings by subsequent MACE occurrences; mean baseline CRP was 23.0 ± 26.0 mg/L in patients who developed MACE and 24.0 ± 29.0 mg/L in those who did not, without further specifying a statistical comparison between groups [35]. 

Studies reporting median hsCRP or CRP values demonstrated more substantial reductions following IL-6i or JAKi therapy. Pierini et al. observed a decrease in median hsCRP from 3.9 mg/L (IQR 0.6-17.7) to 0.5 mg/L (IQR 0.2-1.1) after tocilizumab treatment (p<0.0001) [34]. In the cohort receiving filgotinib, Benucci et al. reported median CRP reductions from 11.8 mg/L (IQR 5.0-18.9) to 4.7 mg/L (IQR 0.2-8.5) at three months (p=0.001), a 60% reduction, and to 2.8 mg/L (IQR 1.2-4.9) at six months (p=0.001), a 76% reduction from baseline [23]. Novikova et al. found that median CRP decreased from 18.7 mg/L (IQR 9.30-44.4) at baseline to 1.7 mg/L (IQR 0.6-4.3) after 12 months of tofacitinib treatment (p<0.001) [33]. Gerasimova et al. reported that after 60 months of tocilizumab therapy, median CRP decreased from 29.0 mg/L (IQR 11.0-80.8) to 0.3 mg/L (IQR 0.1-2.1), corresponding to a 99% reduction (p<0.01) [31]. 

These findings demonstrate that treatment with both IL-6i and JAKi was generally associated with reductions in systemic inflammatory markers, although the magnitude of suppression varied across studies, follow-up durations, and drug classes. 

*Lipoprotein (a)* *(Lp(a))*

Because studies reported lipid parameters using varying summary statistics, results are presented descriptively. Pierini et al. assessed the impact of tocilizumab therapy and observed a reduction in median Lp(a) from 48.0 mg/dL (IQR 21.6-122.4) at baseline to 21.6 mg/dL (IQR 9.6-64.8), a 55% decrease, after three months (p<0.01) [34]. 

Rao et al. evaluated baseline mean Lp(a) levels among patients treated with tocilizumab and stratified patients based on those who went on to develop MACE with those who did not [35]. Baseline mean Lp(a) was lower among patients who later developed MACE, being 31.0 ± 31.3 mg/dL, compared with those who did not develop MACE, being 33.8 ± 33.5 mg/dL, although the authors did not note a statistical comparison between groups. Czókolyová et al. reported that Lp(a) levels decreased from a baseline of 18.12 ± 23.78 mg/dL to 14.75 ± 19.03 mg/dL at six months (p=0.013) and 15.08 ± 20.23 at 12 months (p=0.024) of treatment with tofacitinib [28]. Although IL-6 and JAK inhibition were seemingly associated with reductions in Lp(a) concentrations, the clinical significance of these studies remains unclear, as no consistency between baseline Lp(a) and eventual MACE risk was observed. 

Low-Density Lipoprotein Cholesterol (LDL-C) 

Studies evaluating the impact of IL-6i or JAKi on mean LDL-C reported baseline values ranging from 104.4 mg/dL to 132.6 mg/dL [21-22,28]. From a baseline mean LDL-C of 119.9 ± 30.8 mg/dL, Anyfanti et al. observed that after three months of JAKi therapy (either upadacitinib, tofacitinib, or baricitinib), mean LDL-C increased to 126.0 ± 34.7 mg/dL, a 5.1% increase, although statistically insignificant (p=0.405) [22]. Similarly, Alsulaim et al. reported a baseline mean LDL-C of 104.4 ± 27.0 mg/dL, with a mean LDL-C difference of 15.1 ± 20.5 mg/dL after 18 months (p<0.001) and 12.8 ± 27.5 mg/dL after 24 months (p=0.045) [21]. Czókolyová et al. reported that LDL-C increased from a baseline mean of 132.6 ± 32.1 mg/dL to 141.9 ± 36.7 mg/dL at six months (p=0.039) and 150.8 ± 43.3 mg/dL at 12 months (p=0.003) of treatment with tofacitinib [28]. Dougados et al. reported a dose-dependent increase in LDL-C [29]. At three months, patients receiving baricitinib 2 mg daily experienced a mean LDL-C increase of approximately 7.35 ± 1.55 mg/dL (p ≤ 0.01) relative to minimal change in the placebo group, whereas those receiving baricitinib 4 mg daily demonstrated a larger mean increase of 8.51 ± 1.55 mg/dL (p≤0.001) during the same period. This pattern persisted at six months of therapy (p≤0.01). Despite this dose-dependent relationship, the absolute magnitude of LDL-C change remained modest.

Several studies examined LDL-C laboratory fluctuations with respect to eventual CV outcomes. Rao et al. and Charles-Schoeman et al. reported that patients who developed MACE had higher baseline LDL-C, ranging from 123.3 to 123.7 mg/dL, compared with patients who did not, ranging from 113.9 to 116.0 mg/dL [24,26,35]. Rao et al. further evaluated the mean LDL-C change over 24 weeks of tocilizumab therapy, finding slightly higher increases in patients who would develop MACE, being 22.0 ± 33.1 mg/dL, compared with those who did not, being 18.6 ± 29.8 mg/dL; however, the authors did not report a statistical comparison between groups [35].

Studies reporting median LDL-C levels were also analyzed. Baseline median LDL-C ranged from 111.4 to 131.6 mg/dL [23,33]. Benucci et al. found that patients administered filgotinib had an increase from a median baseline of 111.4 mg/dL (IQR 101.0-134.0) to 121.0 mg/dL (IQR 103.2-134.0) at both three and six months, representing an 7.9% increase, although the authors reported that this finding was insignificant [23]. In contrast, Novikova et al. reported a median decrease in LDL-C, secondary to tofacitinib therapy, from 124.1 mg/dL (IQR 106.3-167.4) at baseline to 107.1 mg/dL (IQR 98.6-143.1) after 12 months, a 13.7% reduction; this finding, too, was reported as insignificant [33]. Gerasimova et al. similarly demonstrated a decrease in LDL-C levels after 60 months of tocilizumab therapy, from a median LDL-C of 131.5 mg/dL (IQR 108.3-139.2) to 116.0 mg/dL (IQR 81.2-162.4), although the authors reported that this finding was insignificant [31].

High-Density Lipoprotein Cholesterol (HDL-C)

Several studies that reported HDL-C metrics demonstrated modest increases following IL-6 or JAK inhibition. For studies reporting mean HDL-C, baseline values ranged from 58.0 to 63.4 mg/dL [21-22,28]. Alsulaim et al. observed that HDL-C levels increased by 5.03 mg/dL at three months (p=0.04) and 5.80 mg/dL at six months (p=0.02) in patients treated with tocilizumab [21]. Interestingly, Czókolyová et al. reported that HDL-C levels initially decreased at six months, from a baseline of 63.4 ± 36.7 mg/dL to 62.6 ± 22.4 mg/dL (p=0.047), but subsequently increased to 64.2 ± 19.7 mg/dL at 12 months (p=0.004) following tofacitinib therapy [28]. Dougados et al. reported a dose-dependent increase in HDL-C [29]. At three months, patients receiving baricitinib 2 mg daily experienced a mean increase in HDL-C of approximately 6.19 ± 0.77 mg/dL (p≤0.001) relative to minimal change in the placebo group, whereas those receiving baricitinib 4 mg daily demonstrated a larger mean increase of 8.12 ± 0.77 mg/dL (p≤0.001) during the same period. This pattern persisted at six months of therapy (p≤0.01). Despite this dose-dependent relationship, the absolute magnitude of HDL-C change remained modest. Rao et al. evaluated baseline mean HDL-C levels among patients treated with tocilizumab and stratified patients based on those who went on to develop MACE and those who did not [35]. Baseline mean HDL-C was lower among patients who later developed MACE, being 54.1 ± 15.5 mg/dL, compared with those who did not, being 58.0 ± 15.5 mg/dL. From baseline to week 24, HDL-C increased by 2.3 ± 11.2 mg/dL in patients who developed MACE and by 3.9 ± 11.7 mg/dL in those who did not, although the authors did not report a statistical comparison between groups.

Studies reporting median HDL-C similarly demonstrated modest overall increases. Novikova et al. reported that from a baseline median HDL-C of 52.2 mg/dL (IQR 41.0-71.9), patients treated with tofacitinib had an HDL-C of 74.6 mg/dL (IQR 51.8-82.4) after 12 months (p=0.049) [33]. Benucci et al. reported a baseline median of 53.0 mg/dL (IQR 45.0-58.0), increasing to 58.5 mg/dL (IQR 45.0-60.0) and 57.0 mg/dL (IQR 45.0-61.8) following three and six months of filgotinib therapy, respectively, although these findings were not reported as significant [23]. Gerasimova et al. similarly observed an increase, from a baseline median HDL-C of 61.9 mg/dL (IQR 50.3-69.6) to 69.6 mg/dL (IQR 50.3-77.3) after 60 months of tocilizumab therapy, although no statistical comparison between groups was provided [31]. Despite variability across studies, HDL-C levels remained largely unchanged following therapy.

Total Cholesterol

Treatment with both IL-6i and JAKi has been associated with mild to moderate increases in total cholesterol. For studies reporting mean total cholesterol, baseline values ranged from 174.0 to 199.1 mg/dL [21-22,26,28]. Anyfanti et al. observed no change in total cholesterol, with mean values remaining at 195.0 mg/dL after three months of JAKi therapy (either upadacitinib, tofacitinib, or baricitinib) (p=0.859) [22]. Alsulaim et al. reported that from a mean baseline of 174.0 mg/dL, total cholesterol increased by 17.8 ± 43.7 mg/dL and 14.7 ± 32.1 mg/dL following tocilizumab therapy at six (p=0.04) and 24 (p=0.047) months, respectively [21]. Czókolyová et al. noted that total cholesterol levels significantly increased from a baseline of 212.2 ± 35.6 mg/dL to 230.1 ± 44.5 mg/dL at six months (p=0.003) and 230.1 ± 46.4 mg/dL at 12 months (p=0.007) of tofacitinib therapy [28].

Rao et al. evaluated total cholesterol changes in patients who did or did not eventually develop MACE after 24 weeks of tocilizumab therapy [35]. The mean increase was slightly higher in patients who developed MACE, being 30.2 ± 37.5 mg/dL, compared to those who did not, being 26.3 ± 35.5 mg/dL, although the statistical comparison between these groups was unclear. 

Studies reporting median total cholesterol fluctuations similarly showed variable changes. Baseline medians ranged from 189.5 to 197.0 mg/dL [23,33]. Benucci et al. reported that total cholesterol transiently decreased from a baseline median of 197.0 mg/dL (IQR 174.0-210.0) to 189.5 mg/dL (IQR 157.0-209.0) following three months of filgotinib therapy before increasing to 203.5 mg/dL (IQR 174.3-211.0) at six months, although the authors reported these findings were not significant [23]. Novikova et al. reported an increase following tofacitinib treatment, with median total cholesterol rising from 196.8 mg/dL (IQR 164.3-247.5) at baseline to 206.3 mg/dL (IQR 180.6-238.6) after 12 months (p=0.08) [33]. Gerasimova et al. similarly observed an increase in total cholesterol from a baseline median of 189.5 mg/dL (IQR 170.1-239.8) to 201.1 mg/dL (IQR 177.9-263.0) following 60 months of tocilizumab therapy, although the authors reported that this finding was insignificant [31]. 

Overall, treatment with IL-6i or JAKi was associated with mild increases in total cholesterol. Although the magnitude of these changes remained variable and modest, the clinical implications remain unclear. Further studies are needed to clarify the implications of these lipid changes and their potential impact on CV risk.

*Major Adverse Cardiovascular Events *(*MACE)*

Because definitions of MACE varied across studies, including differences between three-point and four-point composite endpoints, CV outcomes were extracted and reported according to the definitions used in the original publications. No attempt was made to reclassify individual CV events into a standardized composite endpoint. 

In a large-scale cohort of 3,986 patients receiving intravenous tocilizumab, Rao et al. reported 50 CV events, indicating an overall low MACE burden [35]. Despite generalized elevations in laboratory lipid markers, these changes were not significantly associated with adverse CV outcomes. In fact, the authors reported that greater RA disease activity reduction during therapy was associated with a significantly lower risk of MACE. Aletaha et al. assessed subcutaneous sirukumab at doses of 50 mg (n=605) every four weeks or 100 mg (n=619) every two weeks, with 14 and seven MACE reports, respectively. Thirty-two deaths were reported; however, the CV origin or relationship to IL-6 inhibition was unclear [20]. Gerasimova et al. reported two individual CV events during tocilizumab therapy, including one MI at week 22 and one episode of angina pectoris at week 24, with no CVAs observed [31]. Alsulaim et al. reported a death secondary to acute coronary syndrome in a patient with multiple comorbidities, including hypertension and diabetes [21]. Notably, however, tocilizumab had been held for several months before death due to recurrent infections, emphasizing the highly complex clinical context in which this death took place. In the IL-6i group (n=273; either tocilizumab or sarilumab), Yoshida et al. reported four cases of MACE; these occurrences were further divided into aortic dissection (n=2), acute cardiac insufficiency (n=1), and MI (n=1) [38]. Collectively, these findings suggest that although IL-6 inhibition may cause lipid fluctuations, it is not necessarily associated with a clinically meaningful increase in MACE incidence. 

In contrast, JAKi were associated with a slightly higher absolute frequency of MACE. Charles-Schoeman et al. reported dose-dependent MACE with upadacitinib [24]. Among patients receiving 15 mg (n=3,209), there were 36 MACE, including two nonfatal strokes, one patient having two MIs, and one patient experiencing a nonfatal MI and CV death, as reported by the authors. Patients receiving 30 mg (n=1,204) had 20 MACE occurrences overall, without further specification regarding the type of event. The majority of patients experiencing MACE had two or more CV risk factors at baseline, and no temporal relationship was established between treatment onset and MACE. In another study by Charles-Schoeman et al., phase three studies of tofacitinib administration (n=1,589 for 5 mg twice daily, n=1,611 for 10 mg twice daily, up to 24 months), showed that the 5 mg twice daily group experienced five CV deaths, two MIs, and five CVAs, whereas the 10 mg twice daily group had two CV deaths, three MIs, and five CVAs [26]. In the long term-extension leg of the same study (up to 72 months; n=1,452 for 5 mg twice daily, n=3,375 for 10 mg twice daily), the 5 mg group experienced three CV deaths, four MIs, and five CVAs, whereas the 10 mg group experienced no CV deaths, six MIs, and 14 CVAs. During phase three trials (24-week duration) of barcitinib at 2 mg (n=479) and 4 mg (n=997) doses, Taylor et al. observed one MI, one CVA, and two CV deaths in the 4 mg group, with no MACE in the 2 mg group [37]. Conversely, Dougados et al. reported two MACE occurrences in the placebo group with none in the baricitinib group [29]. Gentileschi et al. observed one MI and one MI with non-obstructive coronary arteries (MINOCA) in patients treated with either tofacitinib, baricitinib, upadacitinib, or filgotinib (n=182) [30]. Smolen et al. evaluated upadacitinib at 15 mg and 20 mg, reporting only one and two MACE, respectively [36]. Cohen et al. also evaluated upadacitinib administered at 15 mg once daily (n=2,630) and 30 mg once daily (n=1,204), reporting 10 CV deaths overall, although the distribution across dosing groups was unspecified [27]. In the JAKi group (n=154; either baricitinib, tofacitinib, upadacitinib, or filgotinib), Yoshida et al. reported 10 cases of MACE; these occurrences were further divided into acute cardiac insufficiency (n=4), cerebral hemorrhage (n=2), subarachnoid hemorrhage (n=2), MI (n=1), or aortic dissection (n=1) [38]. 

Molander et al. reported no MACE events but identified venous thromboembolic events following exposure to IL-6i (tocilizumab or sarilumab; 66 events among 3,019 patients) and JAKi (baricitinib or tofacitinib; 48 events among 2,354 patients), highlighting the need for continued monitoring in this population [32]. 

While JAKi therapy demonstrated higher absolute MACE frequencies compared to studies evaluating IL-6i therapy, the relative event rates remained low and inconsistently reported across studies. Direct comparisons of MACE incidence across studies should be interpreted cautiously, as endpoint definitions and reporting practices differed between trials.

Discussion

The included studies assessed the effects of JAKi or IL-6i on systemic inflammatory markers, circulating cholesterol levels, and overall MACE. The levels of ESR were shown to decrease following a period of use with either IL-6i or JAKi, although the magnitude of change varied among studies [22-23,31,33-34]. Inconsistent results for hsCRP and CRP levels were noted, with one study reporting no change and another showing up to 87.2% decrease after treatment [22,35]. Most studies showed an increase in LDL-C levels following a period of medication use [22-23,28-29,34-35]. Of the studies that measured change in total cholesterol after treatment, all showed an increase [21,23,26,28,33-35]. The incidence of MACE varied across studies and interventions; however, a greater absolute number of events was reported among patients treated with JAK inhibitors. 

Chronic systemic inflammation accelerates catabolism of circulating lipids, impairs HDL-C functionality, and encourages endothelial dysfunction [42]. For this reason, patients with unmanaged RA are at a heightened risk for MACE, despite traditional CV risk markers, such as LDL-C and total cholesterol, appearing low. As disease activity declines, observed with JAKi or IL-6i therapy initiation, lipid concentrations may rise, reflecting restoration of hepatic lipid metabolism rather than true worsening of CV health [43]. 

In this review, both IL-6i and JAKi therapies were demonstrated to actually elicit mild to moderate LDL-C and total cholesterol increases; however, these changes did not consistently translate into higher MACE rates. Meanwhile, the observed declines in ESR and CRP following IL-6i and JAKi therapy emphasize the efficacy that both pharmacological interventions hold in the overall suppression of inflammatory cascades, central to the pathogenesis of RA. Tocilizumab produced pronounced results, reflecting how inhibition of IL-6-mediated acute-phase reactant synthesis contributes greatly to the decrease in systemic inflammation observed in patients with RA [44]. The various JAKi studies also demonstrated efficacious results tied to markers of inflammation, although to a lesser degree. This reduction in inflammatory burden supports the hypothesis that lipid elevations observed post-treatment may represent an inverse corollary to the lipid paradox, where a normalization of lipid metabolism may be secondary to inflammatory control rather than actual pathologic hyperlipidemia. These data highlight the need for additional prospective studies and that sole lipid monitoring is likely insufficient for assessing CV risk in patients with RA being administered targeted immunomodulators. 

Although elevations in total cholesterol and LDL-C levels were variably observed across the evaluated treatment groups, these increases must be interpreted in the context of the patient’s individual inflammatory resolution. The lipid paradox, highlighting that lipid levels and CV events in these patients may be inversely related, demonstrates that even with elevated cholesterol levels, the simultaneous decrease in systemic inflammation may ultimately restore lipoprotein and endothelial function, thus contributing to a reduction in CV risk. In fact, the elevated lipid parameters may signify a transient return to metabolic homeostasis as inflammation continues to subside during these therapeutic regimens. Although this may be the case, the European Alliance of Associations for Rheumatology (EULAR) still recommends empiric management of hyperlipidemia [45]. 

There were numerous limitations of this review. In terms of the methodology, a protocol was not registered before study initiation, potentially limiting transparency. This review also did not include a search of gray literature, such as clinical trial registries, conference abstracts, or government reports, which may have led to the omission of unpublished or non-peer-reviewed data. Pertaining to the databases used, the limited number of studies meeting the inclusion criteria and heterogeneity in study design restricted the generalizability of findings. Laboratory parameters varied substantially across studies, resulting in inconsistent interpretation of inflammatory markers, lipid profiles, and CV outcomes. Although some investigations assessed multiple biomarkers alongside MACE, others focused on isolated variables, limiting cross-study comparability. Additionally, dosing regimens and duration of exposure to JAKi and IL-6i were not uniform, introducing variability in treatment effect estimates. Several studies permitted previous or concurrent use of methotrexate, corticosteroids, other DMARDs, and, in some cases, statins, which may have independently influenced lipid profiles and CV risk. Definitions of MACE also differed modestly between studies, further complicating outcome standardization. Finally, adjustment for traditional CV risk factors, such as sex and age, and modifiable risk factors, such as tobacco use, was inconsistent, thus raising the possibility of residual confounding. 

This review consolidated the present research available on the effects that IL-6i and JAKi have on various aspects of CV health, lipid levels, and overall inflammatory markers. These results demonstrated a need for additional studies to validate findings and could serve as the foundation for regular cardiometabolic screening for patients with RA who are undergoing treatment with these medications. This review also highlighted residual effects that these medications may have on patients, which physicians should consider before choosing the best medicines for their patients. Although these medications are effective in the management of RA, it is important to comprehensively approach the healthcare provided to these patients in a multifaceted fashion.

## Conclusions

Overall, this review suggests that treatment with JAKi and IL-6i in RA is associated with suppression of systemic inflammation and moderate lipid elevations, which may reflect normalization of lipid metabolism following inflammatory control rather than overt CV harm in most populations. However, findings from high-risk groups, including the ORAL Surveillance trial of tofacitinib, indicate increased MACE risk, and heterogeneity across observational studies limits the ability to draw causal conclusions regarding CV safety. Therefore, lipid elevations should be interpreted cautiously and not assumed to reflect benign metabolic normalization. These results highlight that controlling systemic inflammation remains a key determinant of CV outcomes in RA; however, lipid management alone does not fully elucidate the risk. Future research should aim to investigate the mechanistic basis of the lipid paradox, refine CV risk stratification, and integrate rheumatologic and CV data to develop predictive models that accurately reflect the complex interplay between inflammation, lipid alterations, and CV outcomes in patients with RA.

## Appendices

Appendix A 

**Table 5:** Results yielded from EMBASE

EMBASE search strategy		
Search	Keywords	Results
1	rheumatoid arthritis'/exp OR 'rheumatoid arthritis'	310,439
2	‘Il-6 inhibitor*’ OR ‘interleukin 6 inhibitor*’ OR ‘il-6i*’ OR ‘actemra*' OR 'actemra 200*' OR 'atlizumab*' OR 'avtozma*' OR 'bat 1806*' OR 'bat1806*' OR 'biib 800*' OR 'biib800*' OR 'bow 070*' OR 'bow070*' OR 'complarate*' OR 'ct p47*' OR 'ctp47*' OR 'gnr 087*' OR 'gnr087*' OR 'lusinex*' OR 'lzm 008*' OR 'lzm008*' OR 'msb 11456*' OR 'msb11456*' OR 'qx 003s*' OR 'qx003s*' OR 'r 1569*' OR 'r1569*' OR 'rg 1569*' OR 'rg1569*' OR 'ro 4877533*' OR 'ro4877533*' OR 'roactemra*' OR 'temziva*' OR 'tocilizumab aazg*' OR 'tocilizumab anoh*' OR 'tocilizumab bavi*' OR 'tocilizumab-aazg*' OR 'tocilizumab-anoh*' OR 'tocilizumab-bavi*' OR 'tofidence*' OR 'tyenne*' OR 'tocilizumab*' OR 'kevzara*' OR 'kevzara sc*' OR 'regn 88*' OR 'regn88*' OR 'sar 153191*' OR 'sar153191*' OR 'sarilumab*'	36,142
3	jak inhibitor*' OR 'janus kinase inhibitor*' OR 'janus tyrosine kinase inhibitor*' OR 'tofacitinib*' OR 'upadacitinib*' OR 'baricitinib*'	30,872
4	#2 OR #3	62,273
5	#1 AND #4	19,323
6	lipid profile*' OR 'ldl*' OR 'total cholesterol*' OR 'beta lipoprotein' OR 'lipoprotein, beta' OR 'lipoprotein, low density' OR 'lipoproteins, ldl' OR 'low density lipoprotein' OR 'lipid fingerprint' OR 'lipid profiling' OR 'lipidomic fingerprint' OR 'lipidomic fingerprinting' OR 'lipidomic profile' OR 'lipidomic profiling' OR 'lipid fingerprinting' OR 'blood cholesterol' OR 'blood cholesterol level' OR 'cholesterol, blood' OR 'plasma cholesterol' OR 'serum cholesterol' OR 'total blood cholesterol' OR 'cholesterol blood level'	476,692
7	#5 AND #6	751
8	angiocardiopathy' OR 'angiocardiovascular disease' OR 'cardiovascular complication' OR 'cardiovascular diseases' OR 'cardiovascular disorder' OR 'cardiovascular disturbance' OR 'cardiovascular lesion' OR 'cardiovascular syndrome' OR 'cardiovascular vegetative disorder' OR 'complication, cardiovascular' OR 'disease, cardiovascular' OR 'major adverse cardiovascular event' OR 'cardiovascular disease' OR 'cac score' OR 'coronary artery calcification score' OR 'coronary artery calcium scoring' OR 'coronary calcium score' OR 'coronary artery calcium score'	731,487
9	#5 AND #8	1,318
10	#7 AND #9	325

 Appendix B

**Table 6:** Results yielded from Ovid MEDLINE

Ovid MEDLINE search strategy		
Search	Keywords	Results
1	exp rheumatoid arthritis/ or rheumatoid arthritis.mp. [mp=title, book title, abstract, original title, name of substance word, subject heading word, floating sub-heading word, keyword heading word, organism supplementary concept word, protocol supplementary concept word, rare disease supplementary concept word, unique identifier, synonyms, population supplementary concept word, anatomy supplementary concept word]	182,529
2	(‘Il-6 inhibitor*’ OR ‘interleukin 6 inhibitor*’ OR ‘il-6i*’ OR ‘actemra*' OR 'actemra 200*' OR 'atlizumab*' OR 'avtozma*' OR 'bat 1806*' OR 'bat1806*' OR 'biib 800*' OR 'biib800*' OR 'bow 070*' OR 'bow070*' OR 'complarate*' OR 'ct p47*' OR 'ctp47*' OR 'gnr 087*' OR 'gnr087*' OR 'lusinex*' OR 'lzm 008*' OR 'lzm008*' OR 'msb 11456*' OR 'msb11456*' OR 'qx 003s*' OR 'qx003s*' OR 'r 1569*' OR 'r1569*' OR 'rg 1569*' OR 'rg1569*' OR 'ro 4877533*' OR 'ro4877533*' OR 'roactemra*' OR 'temziva*' OR 'tocilizumab aazg*' OR 'tocilizumab anoh*' OR 'tocilizumab bavi*' OR 'tocilizumab-aazg*' OR 'tocilizumab-anoh*' OR 'tocilizumab-bavi*' OR 'tofidence*' OR 'tyenne*' OR 'tocilizumab*' OR 'kevzara*' OR 'kevzara sc*' OR 'regn 88*' OR 'regn88*' OR 'sar 153191*' OR 'sar153191*' OR 'sarilumab*').ab,ti,kf.	8,616
3	(jak inhibitor*' OR 'janus kinase inhibitor*' OR 'janus tyrosine kinase inhibitor*' OR 'tofacitinib*' OR 'upadacitinib*' OR 'baricitinib*').ab,ti,kf.	11,983
4	2 or 3	19,914
5	1 and 4	4,753
6	(lipid profile*' OR 'ldl*' OR 'total cholesterol*' OR 'beta lipoprotein' OR 'lipoprotein, beta' OR 'lipoprotein, low density' OR 'lipoproteins, ldl' OR 'low density lipoprotein' OR 'lipid fingerprint' OR 'lipid profiling' OR 'lipidomic fingerprint' OR 'lipidomic fingerprinting' OR 'lipidomic profile' OR 'lipidomic profiling' OR 'lipid fingerprinting' OR 'blood cholesterol' OR 'blood cholesterol level' OR 'cholesterol, blood' OR 'plasma cholesterol' OR 'serum cholesterol' OR 'total blood cholesterol' OR 'cholesterol blood level').ab,ti,kf.	232,443
7	5 and 6	122
8	(angiocardiopathy' OR 'angiocardiovascular disease' OR 'cardiovascular complication' OR 'cardiovascular diseases' OR 'cardiovascular disorder' OR 'cardiovascular disturbance' OR 'cardiovascular lesion' OR 'cardiovascular syndrome' OR 'cardiovascular vegetative disorder' OR 'complication, cardiovascular' OR 'disease, cardiovascular' OR 'major adverse cardiovascular event' OR 'cardiovascular disease' OR 'cac score' OR 'coronary artery calcification score' OR 'coronary artery calcium scoring' OR 'coronary calcium score' OR 'coronary artery calcium score').ab,ti,kf.	291,945
9	5 and 8	153
10	7 and 9	31

Appendix C 

**Table 7:** Results yielded from Web of Science

Web of Science search strategy		
Search	Keywords	Results
1	TS=("rheumatoid arthritis")	215,279
2	TS=("Il-6 inhibitor*" OR "interleukin 6 inhibitor*" OR "il-6i*" OR "actemra"' OR "actemra 200*" OR "atlizumab*" OR "avtozma*" OR "bat 1806*" OR "bat1806*" OR "biib 800*" OR "biib800*" OR "bow 070*" OR "bow070*" OR "complarate*" OR "ct p47*" OR "ctp47*" OR "gnr 087*" OR "gnr087*" OR "lusinex*" OR "lzm 008*" OR "lzm008*" OR "msb 11456*" OR "msb11456*" OR "qx 003s*" OR "qx003s*" OR "r 1569*" OR "r1569*" OR "rg 1569*" OR "rg1569*" OR "ro 4877533*" OR "ro4877533*" OR "roactemra*" OR "temziva*" OR "tocilizumab aazg*" OR "tocilizumab anoh*" OR "tocilizumab bavi*" OR "tocilizumab-aazg*" OR "tocilizumab-anoh*" OR "tocilizumab-bavi*" OR "tofidence*" OR "tyenne*" OR "tocilizumab*" OR "kevzara*" OR "kevzara sc*" OR "regn 88*" OR "regn88*" OR "sar 153191*" OR "sar153191*" OR "sarilumab*")	11,783
3	TS=(“jak inhibitor*” OR “janus kinase inhibitor*'”OR “janus tyrosine kinase inhibitor*” OR “tofacitinib*” OR “upadacitinib*” OR “baricitinib*”)	16,713
4	#2 OR #3	27,719
5	#1 AND #4	8,258
6	TS=(“lipid profile*” OR “ldl*” OR “total cholesterol*” OR “beta lipoprotein” OR “lipoprotein, beta” OR “lipoprotein, low density” OR “lipoproteins, ldl” OR “low density lipoprotein” OR “lipid fingerprint” OR “lipid profiling” OR “lipidomic fingerprint” OR “lipidomic fingerprinting” OR “lipidomic profile” OR “lipidomic profiling” OR “lipid fingerprinting” OR “blood cholesterol” OR “blood cholesterol level” OR “cholesterol, blood” OR “plasma cholesterol” OR “serum cholesterol” OR “total blood cholesterol” OR “cholesterol blood level”)	265,686
7	#5 AND #6	179
8	TS=(“angiocardiopathy” OR “angiocardiovascular disease” OR “cardiovascular complication” OR “cardiovascular diseases” OR “cardiovascular disorder” OR “cardiovascular disturbance” OR “cardiovascular lesion” OR “cardiovascular syndrome” OR “cardiovascular vegetative disorder” OR “complication, cardiovascular” OR “disease, cardiovascular” OR “major adverse cardiovascular event” OR “cardiovascular disease” OR “cac score” OR “coronary artery calcification score” OR “coronary artery calcium scoring” OR “coronary calcium score” OR “coronary artery calcium score”)	377,919
9	#5 AND #8	226
10	#7 AND #9	49
